# Cystic fibrosis rabbits develop spontaneous hepatobiliary lesions and CF-associated liver disease (CFLD)-like phenotypes

**DOI:** 10.1093/pnasnexus/pgac306

**Published:** 2022-12-23

**Authors:** Qingtian Wu, Xiubin Liang, Xia Hou, Zhenfeng Song, Mohamad Bouhamdan, Yining Qiu, Yui Koike, Carthic Rajagopalan, Hong-Guang Wei, Hong Jiang, Gerry Hish, Jifeng Zhang, Y Eugene Chen, Jian-Ping Jin, Jie Xu, Kezhong Zhang, Fei Sun

**Affiliations:** Department of Physiology, Wayne State University School of Medicine, Detroit, MI 48201, USA; Center for Advanced Models for Translational Sciences and Therapeutics, University of Michigan Medical Center, University of Michigan Medical School, Ann Arbor, MI 48109, USA; Department of Physiology, Wayne State University School of Medicine, Detroit, MI 48201, USA; Center for Molecular Medicine and Genetics, Wayne State University School of Medicine, Detroit, MI 48201, USA; Department of Physiology, Wayne State University School of Medicine, Detroit, MI 48201, USA; Center for Molecular Medicine and Genetics, Wayne State University School of Medicine, Detroit, MI 48201, USA; Center for Advanced Models for Translational Sciences and Therapeutics, University of Michigan Medical Center, University of Michigan Medical School, Ann Arbor, MI 48109, USA; Department of Physiology, Wayne State University School of Medicine, Detroit, MI 48201, USA; Department of Physiology, Wayne State University School of Medicine, Detroit, MI 48201, USA; Department of Physiology, Wayne State University School of Medicine, Detroit, MI 48201, USA; Laboratory Animal Resources, Wayne State University School of Medicine, Detroit, MI 48201, USA; Center for Advanced Models for Translational Sciences and Therapeutics, University of Michigan Medical Center, University of Michigan Medical School, Ann Arbor, MI 48109, USA; Center for Advanced Models for Translational Sciences and Therapeutics, University of Michigan Medical Center, University of Michigan Medical School, Ann Arbor, MI 48109, USA; Department of Physiology, Wayne State University School of Medicine, Detroit, MI 48201, USA; Center for Advanced Models for Translational Sciences and Therapeutics, University of Michigan Medical Center, University of Michigan Medical School, Ann Arbor, MI 48109, USA; Center for Molecular Medicine and Genetics, Wayne State University School of Medicine, Detroit, MI 48201, USA; Department of Physiology, Wayne State University School of Medicine, Detroit, MI 48201, USA

**Keywords:** cystic fibrosis, CF rabbits, CF related liver disease

## Abstract

Cystic fibrosis (CF) is an autosomal recessive genetic disease affecting multiple organs. Approximately 30% CF patients develop CF-related liver disease (CFLD), which is the third most common cause of morbidity and mortality of CF. CFLD is progressive, and many of the severe forms eventually need liver transplantation. The mechanistic studies and therapeutic interventions to CFLD are unfortunately very limited. Utilizing the CRISPR/Cas9 technology, we recently generated CF rabbits by introducing mutations to the rabbit CF transmembrane conductance regulator (CFTR) gene. Here we report the liver phenotypes and mechanistic insights into the liver pathogenesis in these animals. CF rabbits develop spontaneous hepatobiliary lesions and abnormal biliary secretion accompanied with altered bile acid profiles. They exhibit nonalcoholic steatohepatitis (NASH)-like phenotypes, characterized by hepatic inflammation, steatosis, and fibrosis, as well as altered lipid profiles and diminished glycogen storage. Mechanistically, our data reveal that multiple stress-induced metabolic regulators involved in hepatic lipid homeostasis were up-regulated in the livers of CF-rabbits, and that endoplasmic reticulum (ER) stress response mediated through IRE1α-XBP1 axis as well as NF-κB- and JNK-mediated inflammatory responses prevail in CF rabbit livers. These findings show that CF rabbits manifest many CFLD-like phenotypes and suggest targeting hepatic ER stress and inflammatory pathways for potential CFLD treatment.

Significance Statement Cystic fibrosis (CF)-related liver disease (CFLD) is a major extrapulmonary cause of mortality for CF, yet there is no effective medicine, which is at least partially due to the lack of a clinically relevant but also laboratory-friendly animal model. In the present work, we report that CF rabbits manifest many CFLD-like phenotypes. This novel model is expected to find unique opportunities to facilitate translational biomedical research in CFLD.

## Introduction

Cystic fibrosis (CF) is an autosomal recessive genetic disorder caused by mutations in the CF transmembrane conductance regulator (CFTR) gene (1). CFTR is a chloride channel expressed at the apical membranes of epithelial cells lining the airways and other epithelial tissues. The most common mutation found in the Caucasians of Northern European descent is the deletion of the phenylalanine residue at position 508 (F508del), with approximately 90% of CF Caucasian patients in the United States carry one or two alleles of this specific mutation (2).

CF affects multiple vital organs, including the lung, pancreas, intestine, and liver, among others. Approximately 30% to 40% of CF patients develop CF-associated liver disease (CFLD), which is the third most common morbidity and mortality cause of this disease (3, 4). CFLD is progressive and variable individually. Most CFLD cases have mild clinical presentations, but 5% to 10% will develop cirrhosis and require liver transplantation (5). CFTR is expressed in the cholangiocytes, the lining epithelial cells of the bile duct, but not the hepatocytes in human and animal livers (4, 6–8) including rabbits (Fig. S1). In CF subjects, it is generally believed that the loss of CFTR function in the cholangiocytes causes the obstruction of the bile duct, leading to the development of CFLD (9, 10). However, the pathological process and mechanistic basis underlying CFLD remains to be fully investigated. Partially due to such understudied status, there is a lack of treatment options for CFLD. Currently, liver transplantation is the only effective therapy to severe CFLD cases (11), and ursodeoxycholic acid (UDCA) is the only clinically approved drug for treating CFLD yet with limited efficacy (12).

In 2019, the U.S. Food and Drug Administration (FDA) approved Trikafta (referred to as ETI hereafter), a combination of CFTR potentiator Ivacaftor (VX-770) and CFTR correctors Elexacaftor (VX-445) and Tezacaftor (VX-661), which provides benefits especially on the pulmonary functions to most CF patients including those of the F508del mutation (13). Whether ETI has any beneficial effects on the liver of CF patients remain to be systematically studied, although some pre-ETI-era studies suggested that CFTR modulator drugs along (e.g., ivacaftor) or in combination [e.g., lumacaftor (VX-809)-ivacaftor (VX-770)] may provide benefits to the liver of CF patients (14–16). Of concern however, emerging data presented in the 2021 North America CF Conference indicated that patients under ETI develop worsened liver conditions. For example, Vanscoy et al (17) reported that lung function improvement with ETI in their cohort (*n* = 80) was less robust compared to clinical trial results, whereas significant liver enzyme elevation was more common with potentially life-threatening liver injury in one individual. Vogt et al (18) reported that 27% (71 out of 263) patients in their cohort had elevated hepatic panel parameters including bilirubin, aspartate aminotransferase (AST) and alanine aminotransferase (ALT) after taking ETI. Alarmingly, Ratti et al (19) reported that in their cohort of 100 patients who are under ETI treatment for only 1 y, the number of patients met the criteria for metabolic syndrome grew from 5 to 16, a > 200% increase. Most recently, Stylemans et al reported a case of drug induced liver injury under treatment with ETI (20). With the expectation that improved pulmonary function achieved by ETI will significantly elongate the patients’ lifespan, the probable worsened liver conditions may take a significant long-term toll. As such, in the post-ETI era, how to mitigate liver disease is a priority for CF research.

Studies using animal models have made significant contributions to CF research and drug discoveries. To date, six mammalian CF animal models have been reported, including mice, rats, pigs, ferrets, sheep, and rabbits (reviewed in ref (21)). With regard to liver phenotypes, in the small rodent models, CF rats did not show liver problems (22), and CF mice showed liver impairment only at old age (>12 months) under high-fat diet (23). In the large animal models, CF pigs (24), and sheep (25) manifested liver phenotypes; these untraditional model animals, however, are not easily adoptable by most laboratories due to the challenges of housing and care requirements.

We recently reported the development of CF rabbits by CRISPR/Cas9-mediated gene knockout (26). A guide RNA targeting Exon 13 of the rabbit CFTR gene was used. This Exon encodes the nucleotide-binding domain 1 (NBD1) of the protein where many CF causing mutations including F508del reside. Our model (CF-9) carries a 3 amino acid (P477, S478, and E479) deletion in this domain (Fig. S2), which is predicted to cause the protein misfolding and defective trafficking to the membrane. The derived CF rabbits, as expected, develop typical CF phenotypes including liver related disorders (26), therefore holding the potential to serve as a practical model for CFLD study.

In the present work, we report liver related phenotypes in CF rabbits. CF rabbits develop spontaneous hepatobiliary lesions and CFLD-like phenotypes. Our work presents a new CFLD animal model that is both clinically relevant and laboratory friendly for translational and mechanistic studies.

## Results

### CF rabbits develop spontaneous hepatobiliary lesions

We previously reported that CF-9 rabbits exhibit many typical CF phenotypes (26), such as growth retardation, intestinal obstruction, and airway abnormalities. Here we conducted in-depth characterizations of the hepatic biliary system in the liver of CF-9 rabbits (referred to as “CF rabbits” interchangeably hereafter) in a new cohort of animals (Fig. S3).

**Fig. 1.**
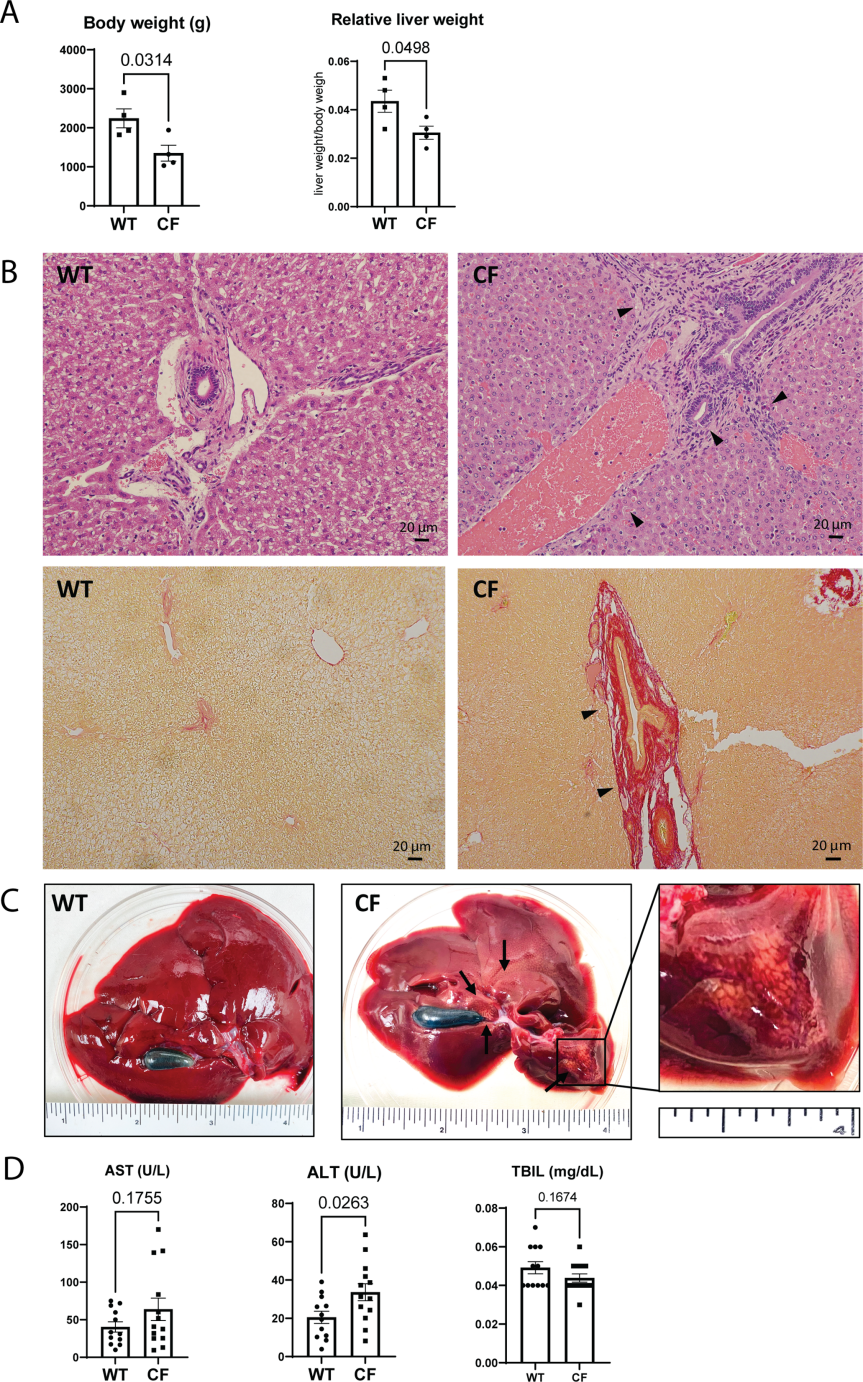
Hepatobiliary lesions in CF rabbits. (A) Body weight and relative liver weight of CF (*n* = 4, 96 ± 11 days of age) and WT (*n* = 4, 91 ± 12 days of age) rabbits. (B) Hematoxylin and eosin (H&E) (upper panels) and Sirius-red staining (lower panels) of liver sections of CF and WT rabbits show typical biliary cirrhosis (arrows) and mucus plugs in the CF rabbits. Scale bars: 20 μm. (C) Representative gross images of WT and CF rabbit livers. The zoom-in box shows representative focal simple macrovesicular steatosis. (D) ALT, AST, and TBIL levels in CF (*n* = 13, 42 ± 2 days of age) and WT (*n* = 12, 45 ± 2 days of age) rabbits.

Consistent with our previous report (26), CF rabbits were of lower body weight compared to age matched nonCF rabbits (Fig. 1A, left). Comparing to wild-type (WT) rabbits, many CF rabbits suffer from inappetite (Fig. S4). The relative liver weights (liver weight/body weight) of these CF rabbits were also significantly lower than those of the controls (Fig. 1A, right).

H&E staining and the Sirius-red staining reveal that CF rabbit livers are associated with focal biliary fibrosis and cirrhosis around the bile duct accompanied with mucus plug (Fig. 1B). Disoriented epithelial cells lining the bile triad ducts as well as stenosis in ducts were found in CF rabbits, but not in WT rabbits. Infiltration of heterophils (the rabbit equivalent of neutrophils) and lymphocytes was noticed only in CF rabbit liver sections. In total we have observed biliary fibrosis in 7 out of 19 (37%) CF rabbits (age between 4 weeks and 7.5 months). Consistently, at the macroscopic level, some CF rabbits (2 out of 4 examined, age 9 weeks old) displayed focal simple macrovesicular steatosis, exampled in Fig. 1C where lesions surrounded the porta hepatis and steatosis on the left lobe were obvious.

We also assessed the serum levels of liver enzymes aspartate aminotransferase (AST), alanine aminotransferase (ALT) and total bilirubin (TBIL) from WT and CF rabbits of approximately 6 weeks old. The levels of ALT in CF rabbits were increased, compared to those in WT control rabbits (Fig. 1D), indicative of liver damage in these CF animals. The other two (ALT and TBIL) were similar between these two groups.

### Abnormal bile acid (BA) secretion and BA profiles in CF rabbits

**Fig. 2.**
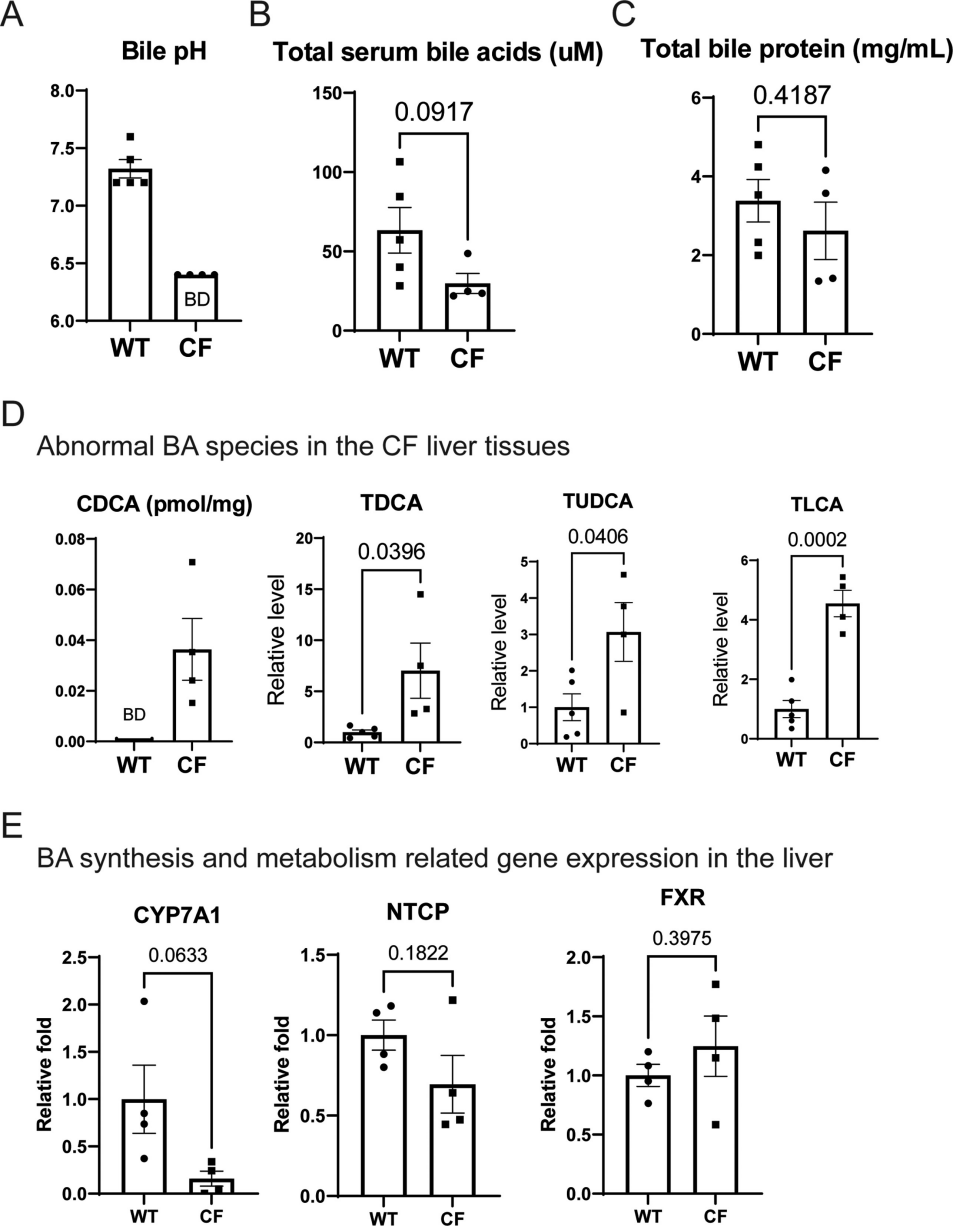
Abnormal biliary secretion in CF rabbits. (A) Bile pH of CF (*n* = 4, 43 ± 3 days of age) and WT (*n* = 5, 43 ± 4 days of age) rabbits. The pH values of CF rabbits all reached below the detection limit (6.4) of the pH paper, thus 6.4 values were used for graphing. BD: below detection limit. (B) Levels of total serum BAs in CF (*n* = 4, 43 ± 3 days of age) and WT (*n* = 5, 43 ± 4 days of age) rabbits. (C) Relative serum bile protein abundance in CF (*n* = 4, 43 ± 3 days of age) and WT (*n*  = 5, 43 ± 4 days of age) rabbits. (D) Abnormal levels of representative BA species in CF rabbit liver. The CDCA levels in WT rabbit liver tissues were below the detection limit (BD) thus zero values were used for graphing. (E) Expression profiles of the genes involved in BA synthesis and metabolism, including CYP7A1, Na/Taurocholate Cotransporting Polypeptide (NTCP), and farnesoid X receptor (FXR) in the livers of WT and CF rabbits, determined by qPCR analysis. The average of all the control expression levels was set as 1, which was used to calculate the fold changes of all the individual expression levels.

We proceeded to examine the BAs in CF rabbits. It is known that BA dysregulation contributes to CFLD (27). One of the first event in CFLD pathogenesis is the reduced bile flow due to the increased bile viscosity (28). Indeed, the bile from all CF rabbits that were examined (*n* = 4, 6 weeks old) was thick and tenacious (Fig. S5A) and exhibited crystal-like pigments (Fig. S5B), while the bile collected from the gallbladders of nonCF [e.g., WT and heterozygous (HT)] rabbits flowed easily. Furthermore, the bile pH of CF rabbits was lower than that of WT (Fig. 2A), consistent with the knowledge obtained from CF animal models (29, 30) and CF patients (28). The levels of total serum BAs in the CF rabbits were lower than that in the WT animals (Fig. 2B), whereas the total bile protein abundance was similar between the CF and WT rabbits (Fig. 2C).

In CF patients, the serum levels of many BA species including cholic acid (CA), chenodeoxycholic acid (CDCA) and others are reportedly upregulated (31). In CF mice, which rarely manifest any liver pathology, some BA species changed in a direction that is opposite from the human data, for example, CA and CDCA were both downregulated in the CF mouse bile in one study (32). In the two models that have liver phenotypes (i.e., CF pigs and CF sheep), there is little reported BA species information yet.

Here we collected bile and liver tissue samples from WT and CF rabbits of 6 weeks old and profiled different BA species in these samples by metabolomics analysis. The target metabolomics analysis showed that many BA species, including both primary and secondary ones are altered in both the bile and the liver tissues of CF rabbits in comparison to that from WT animals (Fig. 2D, Figs. S6 and S7). Particularly, CDCA, taurodeoxycholic acid (TDCA), tauroursodeoxycholic acid (TUDCA) and taurolithocholic acid (TLCA) were significantly elevated in CF rabbit livers, which may represent a pathological response to the development of nonalcoholic steatohepatitis (NASH)-like phenotype in these animals as described later in this report.

We analyzed the expression of the genes involved in hepatic BA biosynthesis and metabolism in the livers of CF and WT control rabbits by quantitative real-time PCR (qPCR) analyses (Fig. 2E). Although the expression levels of Na/Taurocholate Cotransporting Polypeptide (NTCP), a major transporter of hepatic BA (33), and FXR, a transcriptional regulator of BA synthesis and transport (34, 35), were not significantly different between the WT and CF rabbit livers, the expression levels of the transcripts encoding Cytochrome P450 Family 7 Subfamily A Member 1 (CYP7A1), the rate-limiting enzyme mediating BA biosynthesis and metabolism (36), in the CF rabbit livers was significantly reduced, compared to that of the WT rabbit livers (Fig. 2E). These results suggest that CFTR deficiency may impair hepatic BA biosynthesis and metabolism.

### NASH-like phenotypes in CF rabbits

**Fig. 3.**
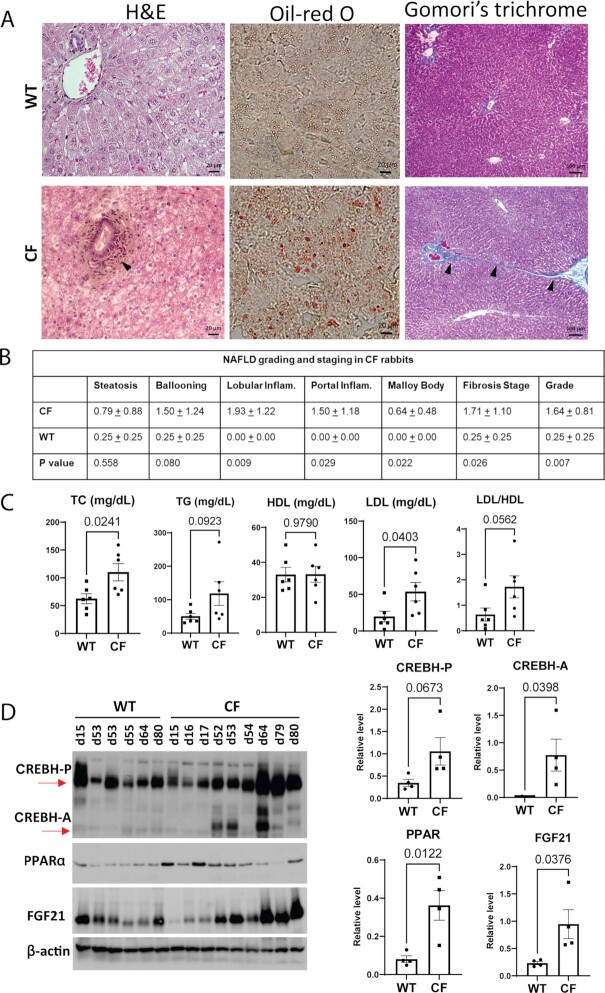
NASH phenotype and lipid disorder in CF rabbits. (A) Histological analysis of liver cellular structure (H&E, left column), lipid accumulation (oil-red O, central column), and collagen fiber (Gomori's trichrome staining, right column) in liver tissue sections from WT and CF rabbits. Arrows point to areas of hepatic inflammation or fibrosis. Scale bars: 20 μm in the left and middle panels; 100 μm in the right panel (B) Scoring for NASH activities in CF (*n* = 14, 50 ± 11 days of age) and WT (*n* = 4, 87 ± 3 days of age) rabbit livers based on the modified Brunt scoring system. (C) Levels of plasma total cholesterol(TC), triglycerides (TG), high-density lipoprotein (HDL), and lipoprotein cholesterol (LDL) in WT (*n* = 6, 50 ± 3 days of age) and CF (*n* = 6, 47 ± 3 days of age) rabbits. (D) Left: Western blot of Cyclic AMP-responsive element-binding protein H (CREBH), peroxisome proliferator-activated receptor α (PPARα) and Fibroblast growth factor 21 (FGF21) protein levels in WT and CF rabbits of different ages (indicated by numbers on the top of the gel, e.g., d15). Right: Quantification of CREBH, PPARα, and FGF21 protein levels in WT and CF rabbits at 50 to 70 days of age. CREBH precursor (CREBH-P): CREBH precursor; CREBH-A: activated CREBH protein.

Many CF patients present with hepatic steatosis and NASH phenotypes (37, 38) and develop metabolic risk factors associated with NASH (39). To assess NASH-associated activities in CF rabbits, we performed histological analyses of liver tissue sections from WT and CF rabbits. Based on H&E staining of liver cellular structure, oil-red O staining of hepatic lipids, and Gomori's trichrome staining of the hepatic collagen deposition, we identified increased hepatic steatosis, lobular and portal inflammation, as well as perisinusoidal and portal fibrosis in the liver of CF rabbits of 7-weeks old as compared to WT controls of 12 weeks old (Fig. 3A). Using the NAFLD (nonalcoholic fatty liver disease) grading and staging score system (40, 41), we confirmed that at this age (7 weeks) greater than 50% CF rabbits (8 out of 14) developed NASH-like phenotype (defined as a “Grade” score of 2 or higher), characterized by steatosis, hepatic inflammation, and/or perisinusoidal/portal fibrosis (Fig. 3B, Table S1).

### Abnormal lipid profiles and up-regulation of hepatic metabolic regulators

In the pre-ETI-era, dyslipidemia is not a primary concern for the CF population. Serum cholesterol concentrations are generally low in CF patients, whereas hypertriglyceridemia appears to be more common (42). In the post-ETI-ear however, the prevalence of obesity in CF is increasing, and around one-third of adults with CF are now overweight or obese (43). There is a prediction that obesity related lipid disorders such as hypercholesteremia may become more common and consequently lead to increased cardiovascular (CV) events in the CF patients. We note that rabbit is a classic animal model to study cardiovascular diseases and associated lipid biology (44–46), thanks to many similarities of CV physiology between humans and rabbits (47). As such, CF rabbits may be a readily available model to study CV diseases in CF in the future.

Here we assessed the lipid profiles of 7-weeks-old CF rabbits in comparison to those of WT. It is revealed that CF animals have abnormally high levels of plasma TG, TC, and low-density LDL, but not HDL (Fig. 3C). Consistently in the liver tissues, the levels of TC, TG, and free fatty acids (FFA) were higher in the CF than in the WT rabbits (Fig. S8). These findings implicate a prevalence of hyperlipidemia consisting of both hypercholesteremia and hypertriglyceridemia in these animals.

To gain mechanistic insights into the lipid phenotype of CF rabbits, we examined the expression of several major metabolic regulators in the rabbit livers. CREBH and PPARα, two binary liver-enriched and stress-inducible transcriptional regulators, play important regulatory roles in hepatic lipid and glucose metabolism (48–51). In comparison to that of WT rabbits, expression levels of CREBH-P, activated CREBH protein (CREBH-A), and PPARα in the livers of CF rabbits were increased in an age-progressive manner (Fig. 3D). FGF21, whose expression is regulated by the CREBH-PPARα transcriptional complex, is a major hepatokine that drives mobilization of lipids and glucose in response to stress challenges (49). The expression levels of FGF21 in CF rabbit livers were increased in an age-progressive manner (Fig. 3D). The upregulation of the CREBH/PPARα/FGF21 regulatory axis may represent a feedback regulation in CF rabbit livers as a compensation for liver function in CFLD.

Additionally, we examined the expression of the genes encoding the major enzymes or regulators in hepatic lipid metabolism in the CF rabbit livers. Interestingly, CFTR deficiency led to significant increases in expression of the transcripts encoding the functions involved in TG lipolysis, including ApoC2 and ApoA4 (Fig. S9A), but not those involved in fatty acid (FA) oxidation, including PPARα, BDH1, Acox1, and CD36 (Fig. S9B). The differential regulation of the genes involved in different metabolic pathways in response to CFTR defect is an interesting question to be investigated in the future. On the other hand, the expression levels of the transcripts encoding Forkhead Box A1 (FOXA1) and Hepatocyte Nuclear Factor 4α (HNF4α), two major transcriptional regulators of liver differentiation, in the livers of CF rabbits were comparable to those in the WT rabbits (Fig. S9C), which indicates that CFTR deficiency does not significantly affect the liver development.

### Diminished hepatic glycogen storage in CF rabbit livers

**Fig. 4.**
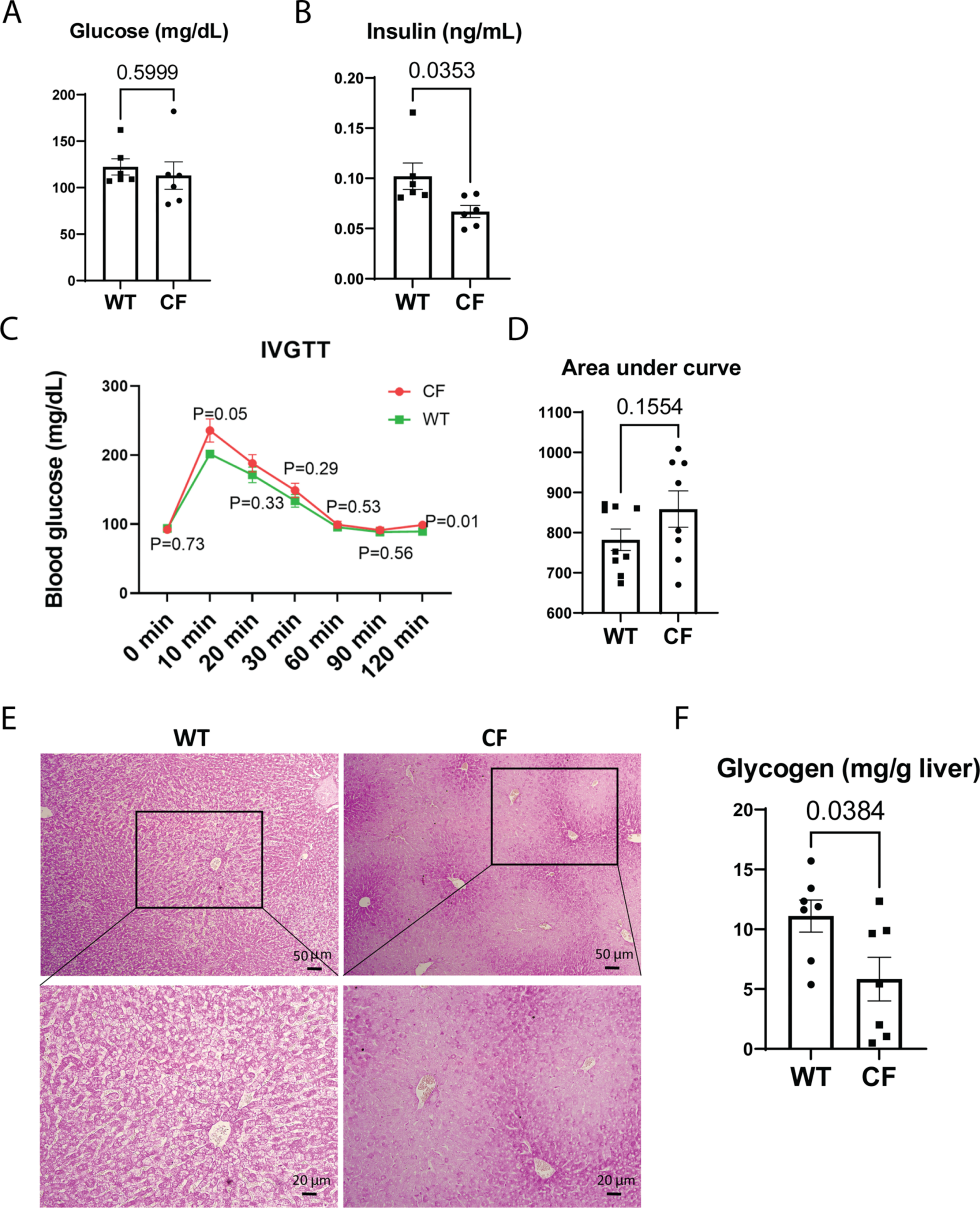
Glucose intolerance in CF rabbits. (A) Levels of fasting blood glucose in CF (*n* = 6, 41 ± 1 days of age) and WT (*n* = 6, 38 days of age) rabbits. (B) Levels of fasting insulin of CF (*n* = 6, 41 ± 1 days of age) and WT (*n* = 6, 38 days of age) rabbits. (C) Intravenous glucose tolerance test (IVGTT) curves of CF (*n *= 8, 49 ± 1 days of age) and WT (*n* = 9, 48 ± 1 days of age) rabbits. *P* values indicate the statistical differences between CF and WT rabbits at each corresponding timepoint. (D) Area under curve values of the IVGTT curves of CF and WT rabbits. (E) Periodic acid-Schiff (PAS) staining of hepatic glycogens in CF and WT rabbits. Boxes in the upper panels correspond to the higher magnification images in lower panels. Scale bars: 50 μm in the upper panels; 20 μm in the lower panels. (F) Enzymatic assay of hepatic glycogens in CF (*n* = 7, 43 ± 2 days of age) and WT (*n* = 7, 41 ± 3 days of age) rabbits.

To assess the manifestation of CFLD in the context of glucose homeostasis, we first examined fasting blood glucose and insulin levels in CF and WT rabbits at 5 to 6 weeks of age. The levels of blood glucose were similar (Fig. 4A), while blood insulin levels were decreased in CF rabbits compared to that of WT controls (Fig. 4B). Random blood glucose measurements of animals of older ages indicated that many CF rabbits older than 3 months of age had elevated blood glucose levels (Fig. S10A).

Next, we performed IVGTT in WT and CF rabbits of approximately 7 weeks old. Compared to WT controls, CF rabbits exhibited signs of impaired glucose tolerance (Fig. 4C and D) although the differences were not statistically significant at this age.

Insulin tolerance test (ITT) showed that despite the overall lower insulin levels (Fig. 4B) CF rabbits at this age do not suffer from insulin resistance (Fig. S10B). Confirming the results, the values of homeostatic model assessment of insulin resistance (HOMA-IR) were lower in CF animals than that in WT animals, which was caused by the low insulin levels, whereas the values of quantitative insulin-sensitivity check index (QUICKI) were similar between CF and WT rabbits (Fig. S10C).

Given the central role of hepatic glycogen storage in glucose homeostasis, we examined the levels of hepatic glycogen in WT and CF rabbits. Strikingly, many CF rabbits of 5 to 6 weeks age displayed diminished hepatic glycogen storage, as indicated by the PAS staining and confirmed by the quantitative enzymatic assay (Fig. 4E and F, Fig. S10D). The glycogen insufficiency is also suggested by the compromised glucose neogenesis of CF rabbits revealed in the ITT test (Fig. S10B).

### ER stress and inflammatory responses prevail in CF rabbit livers

**Fig. 5.**
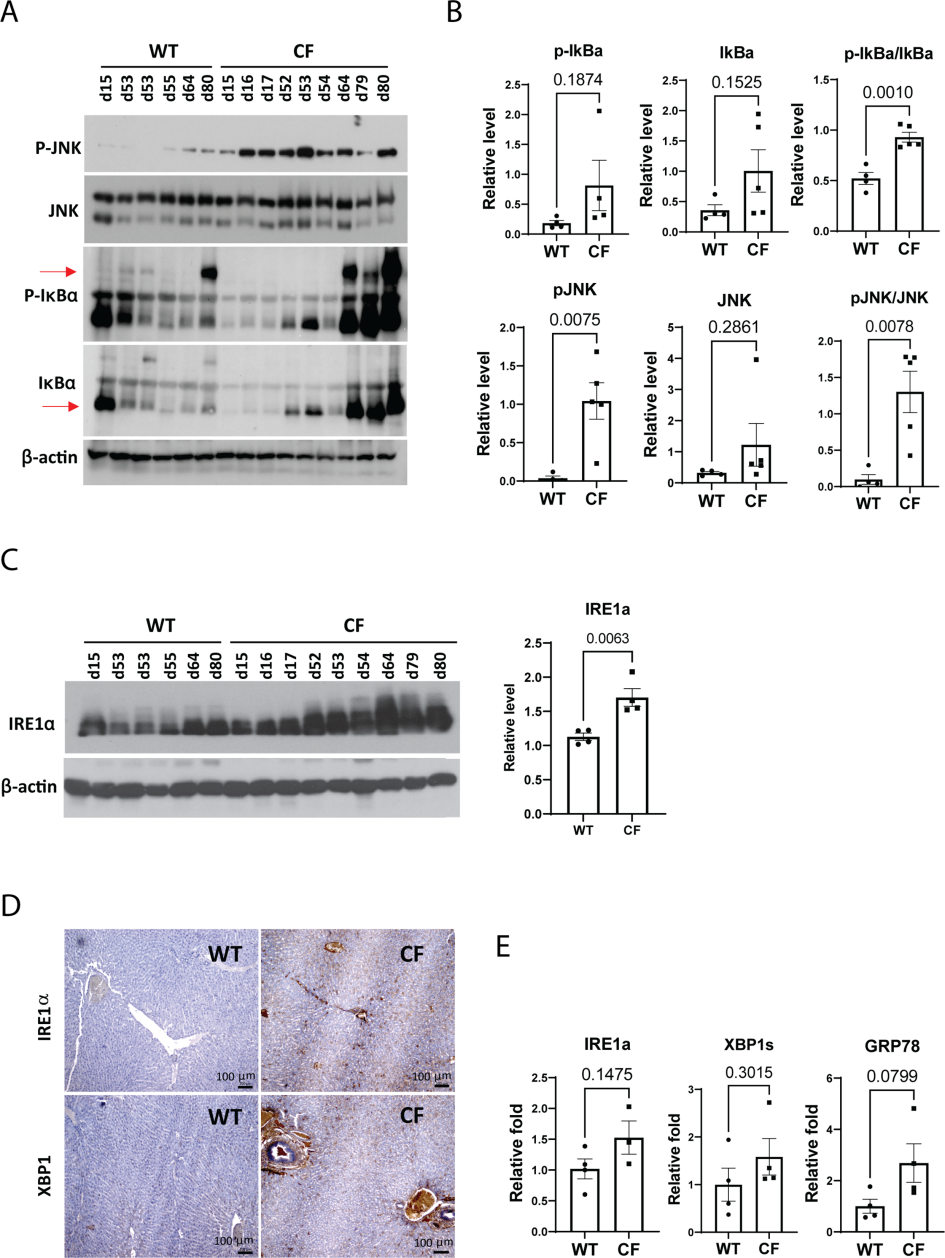
Inflammation and ER stress signaling in CF rabbit livers. (A) Western blot analyses of phosphorylated JNK (P-JNK), total JNK, phosphorylated IκB (P-IκB), and total IκB protein in WT and CF rabbits of different ages (indicated by numbers on the top of the gel, e.g., d15). (B) Quantification of Western blot results of phosphorylated JNK (P-JNK), total JNK, phosphorylated IκB (P-IκB) and total IκB protein in WT and CF rabbits at 50 to 70 days of age. (C) Left: Western blot analyses of IRE1α protein levels in WT and CF rabbits of different ages (indicated by numbers on the top of the gel, e.g., d15). Right: Quantification of Western blot results of IRE1α protein in WT and CF rabbits at 50 to 70 days of age. (D) IHC staining of IRE1α and XBP1 with liver sections from WT and CF rabbits of around 60 days old. Scale bars: 100 μm. (E) qPCR analyses of the mRNAs encoding ER stress sensor or mediators in the livers of WT and CF rabbits at 50 to 70 days of age.

To understand the mechanistic basis underlying the CFLD phenotypes of CF rabbits, we examined the activation of the major inflammatory pathways mediated by JNK and NF-κB in CF rabbit livers. Compared to the WT rabbits, levels of phosphorylated JNK (P-JNK) and phosphorylated NF-κB inhibitor (P-IκB), the indicators of JNK- and NF-κB-mediated inflammatory pathways, were increased in the livers of CF rabbits in an age-progressive manner (Fig. 5A and B).

Next, we investigated activation of the ER stress response, an intracellular stress signaling that promotes inflammation and remodels metabolic homeostasis (52, 53), in the livers of CF and WT control rabbits. Western blot analysis revealed age-dependent activation of ER stress response mediated by the primary ER stress sensor IRE1α in CF rabbit livers (Fig. 5C). Immunohistochemistry (IHC) staining of rabbit liver tissue sections detected strong induction of IRE1α and its downstream transcriptional activator XBP1 around the hepatic biliary ducts of CF but not WT rabbits of about 60 days of age (Fig. 5D). The activation of ER stress responses through IRE1α and XBP1 was confirmed by quantitative real-time PCR (qPCR) analyses, which showed a tendency of increase, although not statistically significant, of the mRNA expression levels of IRE1α, spliced Xbp1 (Xbp1s) and ER chaperone BiP/GRP78 (Fig. 5E). Together, these results revealed activation of ER stress and inflammatory responses in the livers of CF rabbits.

## Discussion

CFLD is a common nonpulmonary cause of mortality in CF, affecting about one third CF patients (54). The peak of CFLD is in the pediatric population, but a second wave of liver disease in CF adults has been reported in the past decade in association with the increase in the life expectancy of CF patients (55). To date, UDCA is the only medicine that has gained FDA approval for treating CFLD, yet its efficacy remains controversial (56). ETI, the primary medicine prescribed to CF patients today, has not been fully evaluated for its long-term effects on CFLD. Of concern, emerging data suggest that it may worsen the liver conditions of CF patients (17–20). With these, CFLD is now a priority subject for CF research in the postETI-era (57).

Clinical manifestations of CFLD are heterogeneous, including cholestasis, focal biliary cirrhosis, and steatosis. The disease is asymptomatic in the early stages. Blood parameters have poor predicting accuracy, although abnormal liver enzyme levels such as ALT and AST are often presented in CF patients (58). Liver ultrasound is a key examination in the diagnosis of CFLD, which aids the physicians to evaluate the liver, gallbladder, biliary tree, and importantly, the signs of portal hypertension, which often occurs in late stage CFLD (58). The main histological presentation of CFLD is focal biliary fibrosis, with the primary hypothesis being that the loss of CFTR in the cholangiocytes leads to increased viscosity of the bile, reduced bile flow, and biliary obstruction, and ultimately peribiliary inflammation and fibrosis (58).

An animal model that recapacitates the key pathogenesis axis of CFLD would greatly facilitate the study of the disease. Here we demonstrate the presence of this axis in CF rabbits: the animals have bile duct obstruction, suffer from abnormal BA secretion, including increased bile viscosity, and present with spontaneous hepatobiliary lesions, including focal biliary fibrosis and cirrhosis. We want to point out that CF rabbits are the smallest animal model that manifest these CFLD hallmarks. The smaller models, CF mice and rats, show few liver problems (22, 23). The larger models, CF pigs and sheep, manifest liver phenotypes (24, 25) but are not easily adoptable to a conventional animal research setting. On the other hand, rabbit is a classic model species (59) that is routinely used in most research institutes. Although smaller than pigs and ferrets, rabbits are much larger than the rodents, hence would allow some otherwise inhibitory procedures/measurements, for example liver ultrasound exam, to be conducted in CF rabbits, that should be considered another advantage of this model.

As expected, the liver pathology led to metabolic disorders in CF rabbits. These include: (i) markedly altered BA species in the liver tissue and the bile fluid; (ii) elevated serum and liver TG, TC, and LDL; (iii) diminished hepatic glycogen storage; (iv) signs of glucose intolerance. In our previous work, we reported intestinal dysbiosis in CF rabbits, including altered microbiome abundance involved in glycan degradation (60). These findings together suggest that CF rabbits may serve for the study of liver-related metabolic disorders in CF.

To our knowledge, the present work is the first to comprehensively measure the levels of major BA species in a nonmurine CF animal model, despite that the disruption of BA homeostasis is one major gastrointestinal phenotype of CF (27). Several BA species are found elevated in CF rabbit liver tissues, which include CDCA, a primary BA that is also found upregulated in the CF patient serum. It should be noted that CDCA is a hydrophobic BA, which many consider as a key insult to induce hepatic injury through ER stress and mitochondrial signaling pathway in liver diseases in general (61). Our work thus suggests that CDCA may be a therapeutic target in treating CFLD.

With regard to glucose metabolism, our previous work (26) reported the presence of pancreatic lesions in CF rabbits, suggesting this model will likely develop hence serve as a model for CF-related diabetes (CFRD). Here we demonstrate that diminished hepatic glycogen storage capacity may also play a role in the pathogenesis of CFRD. Interestingly, in these animals (5 to 6 weeks old), we found that the fasting serum insulin levels were lower in the CF rabbits than those of the WT, whereas the fasting serum glucose levels were not different yet. This suggests that insulin deficiency precedes the altered glucose level, thus suggest that serum insulin level might serve as a diagnosis parameter for early CFRD. Nevertheless, a follow up work is warranted to evaluate CFRD-related phenotypes in the CF rabbits, as current work focuses on the liver.

While many CF rabbits developed hypertriglycemia, a common lipid disorder in CF patients, we note that the cholesterol profiles of the CF rabbits differ from those of human patients. In CF patients, the frequency of hypercholesterolemia (<20%) is much lower than that of hypocholesterolemia (up to 50%) (62). In CF rabbits, however, the majority are associated with elevated cholesterol levels. This of course reminds us that no animal model is perfect. But on the other hand, CF rabbits may be suitable for modeling the disease in the subpopulation of CF patients with hypercholesterolemia. A forward perspective is that in the post-ETI-era with continuously improved BMI of CF patients, it is possible that the hypercholesterol phenotype becomes more common. In fact, obesity, which is often associated with hypercholesterol, has emerged as a concern in CF.

Together, we demonstrate that CF rabbits manifest many CFLD-like phenotypes, hence adding this novel model to the toolbox for CFLD research (Table S4). We expect that the CF rabbits serve as a useful model for the basic and translational studies of CFLD.

As an example to facilitate the basic mechanistic study, we observed marked ER stress signals in the CF rabbit liver. These signals are detectable throughout the liver section, including in the hepatocytes, and very strong in the bile duct area presumptively in the cholangiocytes (Fig. 5D). It is known that ER stress plays a role in the CF lung disease (63), but whether it is also present in extrapulmonary organs are not well known. Our data undoubtedly pin ER stress as one potential pathogenesis driving force in CFLD. At the cell type level, in the cholangiocytes where CFTR is expressed, the prolonged expression of misfolded CFTR could trigger the unfolded protein response (UPR), a major form of ER stress. In the hepatocytes where CFTR is not expressed, the ER stress is likely a secondary response in CF conditions. Ribeiro and Boucher previously reported that in the F508del airway epithelial cells inflammation leads to the activation of XBP-1 and consequently ER stress (63). We speculate that the same may have happened to the hepatocytes, given the elevated inflammation markers observed in the CF rabbit liver. Nevertheless, follow-up studies are needed to elucidate the roles of ER stress in different cell types in CFLD.

We also expect CF rabbits contribute to translational studies. For example, the observation of marked ER stress signals in CF rabbit liver suggests that targeting these pathways may alleviate CFLD. In support of this notion, UDCA, the only FDA approved medicine to treat CFLD, is an effective ER stress inhibitor (37, 64). TUDCA, the taurine-conjugated UDCA, has gone through a Phase II clinical trial for CFLD. Like UDCA, TUDCA has been shown to improve ER homeostasis and reduce ER stress response associated with hepatic steatosis, inflammation, and ER stress-associated apoptosis (45–47). In this regard, CF rabbits would serve well to test new ER stress modulators treating CFLD. Similarly, the upregulation of the JNK pathway in the CF rabbit liver would suggest the testing of JNK inhibitors for treating CFLD. In yet another example, as NASH-like phenotypes such as hepatic steatosis, inflammation and fibrosis are observed in ∼50% CF rabbits, medications developed toward NASH may also benefit CFLD.

We want to point out several limitations of the present study. First, the CF-9 mutation is an artificial mutation created by CRISPR/Cas9, which is not reported in CF patients. Although, we predict this mutation is similar to that of F508del, it is important to evaluate and confirm the findings in our newly developed F508del rabbits (65). Second, the disease phenotypes are relatively consistent in the cohort of animals (e.g., more than half showed NASH-like phenotypes), which differs from the huge variation of disease manifestation of CFLD in human patients. This is likely due to the relatively inbred genetic background of these experimental animals. Nevertheless, this could be exploited as an advantage for the study of the severe forms of CFLD.

In sum, the present work shows that CF rabbits manifest many CFLD-like phenotypes and may serve as a novel model system for the research and development of CFLD.

## Materials and Methods

### Animals

WT and CF rabbits were used in the study. The CF rabbits were developed and maintained as described in a previous study (26). All animal procedures were approved by the Institutional Animal Care and Use Committee (IACUC) of the Wayne State University (WSU) and the University of Michigan (UM) and were performed in accordance with the institutional guidelines.

In total 68 CF rabbits were used: 32 at WSU and 36 at UM. For each experiment, a minimal *n* = 3 CF rabbits were assigned. The animal assignment to each assay is illustrated in Fig. S3.

### Materials

Chemicals were purchased from Sigma–Aldrich (St. Louis, MO, USA) unless indicated otherwise. Antibodies against phosphorylated JNK, total JNK, phosphorylated IκBα, total IκBα, PPARα, and CREBH were purchased from Santa Cruz Biotechnologies, Inc. (Santa Cruz, CA, USA). Antibodies against GRP78, IRE1a, Xbp1s, β-actin and the secondary antibodies were purchased from Cell Signaling Technologies (Danvers, MA, USA). Antibodies against FGF21 were purchased from Abcam (Boston, MA, USA). The kit for determining ALT, AST, and FFA were purchased from Abcam. The kit for determining TG, TC, and HDL weaspurchased from Fujifilm Wako Diagnostics USA (Mountain View, CA, USA). The glycogen enzyme-linked immunosorbent assay (ELISA) kit was purchased from BioAssay Systems (Hayward, CA, USA). The rabbit insulin ELISA kit was purchased from Crystal Chem (Elk Grove Village, IL, USA). The periodic acids staining kit, Gomori's trichrome staining kit were purchased from Fisher Scientific (Hampton, NH, USA). The assay kits and antibodies information are listed in Table S2.

### Histology

#### H&E, PAS, sirius-red and gomori trichrome staining

Liver tissues from WT and CF rabbits were fixed with 4% neutral paraformaldehyde and embedded in paraffin. The tissues were sectioned at 5 μm thick and subjected to H&E, PAS, Sirius-red or Gomori's trichrome staining as described previously (66).

#### Oil-red O staining

Frozen liver tissue sections were prepared for Oil-red O staining of lipid contents according to standard protocol (66). Briefly, frozen liver tissue sections of 8 μm were air-dried, and then fixed in 10% formalin. The fixed sections were rinsed in 60% isopropanol followed by staining with freshly prepared Oil-red O solution for 15 minutes. After Oil-red O staining, liver sections were rinsed in 60% isopropanol followed by washing with water before microscopic studies.

#### Histological scoring for NASH activities

Hepatic steatosis, hepatocyte ballooning, lobular and portal inflammation, Mallory bodies, and fibrosis were examined and scored according to the modified Brunt scoring system for NAFLD (40, 67). The grade scores were calculated based on the scores of steatosis, hepatocyte ballooning, lobular and portal inflammation, and Mallory bodies in randomly chosen 10 high-power field. The stage scores of each field were based on the liver fibrosis. The 0 to 3 grading includes: 0, none; 1, mild; 2, moderate; and 3, severe. The fibrosis stages were determined based on the 0 to 4 stage system: 0, none; 1, zone 3 perisinusoidal fibrosis; 2, zone 3 perisinusoidal fibrosis plus portal fibrosis; 3, perisinusoidal fibrosis, portal fibrosis, plus bridging fibrosis; and 4, cirrhosis.

### Measurement of hepatic glycogen

Biochemical quantification of hepatic glycogens in the liver tissues was performed according to the standard protocol (68). Animals (without overnight fasting) were humanized euthanized. Approximately 100 mg of liver tissue from the same liver lobe region of each rabbit were homogenized in ice-cold citrate buffer (0.1 M, pH 4.2). The tissue homogenates were immediately subjected to glycogen measurement using a commercial glycogen assay kit (Table S2) following the manufacturer's instruction. Levels of hepatic glycogens were presented after normalization to liver tissues mass.

### Lipid and serum biochemistry analysis

Whole blood was collected in a red-topped Vacutainer tubes and kept at room temperature for 30 minutes. After centrifugation at 2,000 *g* for 10 minutes, the serum layer was aliquot into 1.5 mL tubes and stored at −80°C. The TG, TC, and HDL were measured using ELISA kits according to the manufactures’ instructions (Table S2). The levels of serum glycogen, insulin, ALT and AST were tested using ELISA kits (Table S2) separately according to the standard protocols. The serum levels of TBIL in rabbits was performed by the In-Vivo Animal Core at the University of Michigan.

### Hepatic TG, TC, and FFA analyses

To assess liver TG, TC, and FFA, lipids from 10 mg liver were extracted in 200 μl of chloroform: isopropanol: NP-40 (7:11:0.1), as previously described (69). Hepatic TG, TC, and FFA levels were determined using commercially available kits (Table S2) according to the manufacturers' instructions.

### Bile protein measurement and BA analysis

Total protein concentration in the bile fluid was tested using a Synergy H1 hybrid reader for absorbance at 280 nm. For BA analysis, 50 mg liver tissue, and 50 μL bile from each rabbit were submitted to Metabolomics Core at the University of Michigan. The methods of BAs analysis in biological fluids and tissues were performed as described earlier (70).

### Determination of blood glucose and insulin levels

Blood glucose was measured with an Elite Glucometer (Bayer). Insulin levels were determined using an insulin ELISA Kit (Table S2). To measure the fasting glucose and insulin levels, animals were fasted for 12 hours. Random glucose were assayed without fasting the animals. In the IVGTT assay, the animals were fasted for 12 hours followed by intravenous injection (i.v.) of glucose (0.5 g/kg body weight). Blood glucose was measured at 0, 10, 20, 30, 60, 90, and 120 minutes after the injection. The areas under the curve (AUC) were calculated by summing the areas of successive trapezoid under the graph. Insulin resistance was assessed via HOMA-IR and QUICKI as described earlier (71, 72). Specifically, HOMA-IR and QUICKI were calculated using the following formula

$$\begin{eqnarray*}
HOMA - IR &=& fasting\,\,glucose ({mg/dl})\nonumber\\
&&\quad \times 24 \times fasting\,\,insulin ({ng/mL})/405
\end{eqnarray*}$$

$$\begin{eqnarray*}
QUICKI &=& 1/\left[ {log({I0}) + log({G0})}\right],\nonumber\\
&&\quad where\,\,I0\,\,is\,\,fasting\,\,insulin\,\,(mU/mL\,\,microunits\,\,per\,\,milliliter)\nonumber\\
&&\quad and\,\,G0\,\,is\,\,fasting\,\,glucose\,\, ( {mg/dl\,\,milligrams\,\,per\,\,decaliter})
.
\end{eqnarray*}$$

### Western blot analysis

Liver tissues collected from WT and CF rabbits were homogenized using an electric homogenizer followed by centrifugation at 4°C. The suspension was frozen at −80°C until analysis. Total protein concentration was determined by the Bradford Protein Assay Reagents (Bio-Rad, USA). 40 µg total protein per sample was used for Western blotting. Blotted membranes were incubated overnight at 4°C with the appropriate primary antibodies (Table S2). After washing, the membranes were incubated with the secondary antibody for 2 hours. After three times of wash, the reactive bands were visualized by incubation with enhanced chemiluminescence substrates and exposure to an X-ray film. The signal intensities were determined by Quantity One (Bio-Rad Life Science, Hercules, California, CA, USA).

### IHC staining

IHC staining was performed on the liver sections (5 μm) after fixation in 4% paraformaldehyde in PBS. As described previously (73), the slides were placed in 0.01 M citrate buffer (pH 6.0) in a pressure cooker (95°C, 2 minutes) to retrieve antigenic epitopes, washed in PBS, and then incubated with anti-IRE1a and anti-XBP1 antibodies (Table S2) overnight at 4°C. After three 5-minute washes with PBS, the sections were incubated with the IHC secondary antibody (Table S2) at 37°C for 30 minutes. The sections were stained with DAB reaction and sealed on slides with neutral glue.

### Quantitative real-time RT-PCR analysis

For real-time PCR, the reaction mixture containing cDNA template, primers, and SYBR Green PCR Master Mix was run in a 7500 Fast Real-time PCR System (Applied Biosystems, Carlsbad, CA, USA). The sequences of real-time PCR primers used in this study are shown in Table S3. Fold changes of mRNA levels were determined after normalization to internal control GAPDH RNA levels.

### Statistics analysis

Data are expressed as mean ± SEM and were analyzed and compared using unpaired, 2-tailed Student's *t* test (Graphpad Prism 9.2.0, San Diego, CA, USA). Statistical significance with *P* < 0.05 is considered significant.

## Supplementary Material

pgac306_Supplemental_File

## Data Availability

The authors confirm that the data supporting the findings of this study are available within the article and its supplementary materials.

## Notes

Classification: Major: Biological Sciences; Minor: Genetics; Physiology
