# Supplementary material for: Cystic fibrosis rabbits develop spontaneous hepatobiliary lesions and CF-associated liver disease (CFLD)-like phenotypes
Source: PNAS Nexus. 2022 Dec 23;2(1):pgac306. doi: 10.1093/pnasnexus/pgac306 (PMC9832953; doi:10.1093/pnasnexus/pgac306)
Supplement: pgac306_Supplemental_File [file pgac306_supplemental_file.zip › PNASNEXUS-PNASNEXUS-2022-00481-s01.docx]

**Supplementary Tables**

**Table S1.** NAFLD scores for individual WT and CF rabbits. Red colored Grade scores are 2 or higher, which are considered as signs of NASH-like phenotypes.

| **ID#** | **Age (d)** | **Genotype** | **Scores** | | | | | | |
| --- | --- | --- | --- | --- | --- | --- | --- | --- | --- |
|  |  |  | Steatosis | Ballooning | Lobular Infacion | Portal Infacion | Malloy Body | Fibrosis Stage | **Grade** |
| W1 | 36 | WT | 0 | 0 | 0 | 0 | 0 | 0 | **0** |
| W2 | 43 | WT | 0 | 0 | 0 | 0 | 0 | 0 | **0** |
| W3 | 80 | WT | 0 | 1 | 0 | 0 | 0 | 0 | **1** |
| W4 | 190 | WT | 1 | 0 | 0 | 0 | 0 | 1 | **0** |
|  |  |  |  |  |  |  |  |  |  |
| CF1 | 35 | CF | 0 | 3 | 3 | 3 | 1 | 3 | **2** |
| CF2 | 19 | CF | 1 | 3 | 3 | 3 | 1 | 4 | **3** |
| CF 3 | 35 | CF | 1 | 0 | 0 | 0 | 0 | 0 | **0** |
| CF 4 | 39 | CF | 0 | 3 | 2 | 2 | 1 | 2 | **2** |
| CF 5 | 41 | CF | 1 | 3 | 3 | 2 | 1 | 2 | **2** |
| CF 6 | 43 | CF | 3 | 2 | 3 | 1 | 1 | 2 | **2** |
| CF 7 | 43 | CF | 0 | 1 | 3 | 3 | 1 | 2 | **3** |
| CF 8 | 43 | CF | 0 | 1 | 3 | 3 | 1 | 2 | **2** |
| CF 9 | 52 | CF | 0 | 1 | 1 | 1 | 0 | 2 | **1** |
| CF 10 | 56 | CF | 0 | 1 | 3 | 2 | 1 | 2 | **1** |
| CF 11 | 64 | CF | 2 | 3 | 2 | 1 | 1 | 2 | **2** |
| CF 12 | 195 | CF | 1 | 0 | 1 | 0 | 0 | 1 | **1** |
| CF 13 | 18 | CF | 1 | 0 | 0 | 0 | 0 | 0 | **1** |
| CF 14 | 23 | CF | 1 | 0 | 0 | 0 | 0 | 0 | **1** |

**Table S2.** Reagents and antibodies used in the present study.

| **Assay Kits** |  |  | Cat# | Conditions |
| --- | --- | --- | --- | --- |
|  | Rabbit insulin | Crystal Chem | 90186 |  |
|  | Glycogen | BioAssay Systems | E2GN-100 |  |
|  | ALT | Abcam | ab234578 |  |
|  | AST | Abcam | ab263881 |  |
|  | Triglyceride | Wako | 290-63701 |  |
|  | Total Cholesterol | Wako | 999-02601 |  |
|  | HDL-Cholesterol | Wako | 997-01301 |  |
|  | Free Fatty Acid Assay | Abcam | ab65341 |  |
|  | PAS Staining | Fisher Scientific | M1016460001 |  |
|  | Gomori’s trichrome | Fisher Scientific | 23-900-662 |  |
| **Antibodies** |  |  |  |  |
| Primary |  |  |  |  |
|  | GRP78 | CST | 3177 | 1:1000 western |
|  | IRE1a | CST | 3294 | 1:1000 western  1:100 IHC |
|  | Xbp1s | CST | 27901 | 1:100 IHC |
|  | p-JNK | Santa Cruz | sc-6254 | 1:1000 western |
|  | JNK | Santa Cruz | sc-7345 | 1:1000 western |
|  | p-IκBα | Santa Cruz | sc-8404 | 1:1000 western |
|  | IκBα, | Santa Cruz | sc-1643 | 1:1000 western |
|  | PPARα | Santa Cruz | sc-398394 | 1:1000 western |
|  | CREBH | Kerafast | EWS101 | 1:1000 western |
|  | FGF21 | Abcam | ab171941 | 1:1000 western |
|  | β-actin | CST | 3700 | 1:1000 western |
| Secondary |  |  |  |  |
|  | Anti-mouse IgG | CST | 7076 | 1:5000 western  1:1000 IHC |
|  | Anti-rabbit IgG | CST | 7074 | 1:5000 western  1:1000 IHC |

**Table S3.** qPCR primer sequences**.**

| Rabbit IRE1α F | ATTGTGTACCGGGGCATGTT |
| --- | --- |
| Rabbit IRE1α R | CTCGTCTGATTCTCGCAGCA |
|  |  |
| Rabbit XBP1s F | GGGGATGGATGCCATGGTTA |
| Rabbit XBP1s R | GCTGCAGATGCACGTAGTCT |
|  |  |
| Rabbit GRP78 F | TGGGTGGTGGAACCTTTGAT |
| Rabbit GRP78 R | TGACACGCTGGTCGAAGTC |
|  |  |
| Rabbit CYP7A1 F | TGGGTGACAGAGGGGATGTA |
| Rabbit CYP7A1 R | GTGCGTCTTGCCTTGTAAGC |
|  |  |
| Rabbit NTCP F | CCTGAAGTCATTGGACCGCT |
| Rabbit NTCP R | GCACCGAAAAACAGCGATGA |
|  |  |
| Rabbit FXR F | CAGCCCGAGAATCCTCAACA |
| Rabbit FXR R | ACTTGTGGTCGTTGACCCTC |
|  |  |
| Rabbit ApoA4 F | CAGACCACGTTCCACGAGAA |
| Rabbit ApoA4 R | TTGTCGGCAAAGGACGTGAG |
|  |  |
| Rabbit ApoC2 F | TGTCCAGCTACTGGGACTCA |
| Rabbit ApoC2 R | GCTCTTGCTGTACATGTCCCT |
|  |  |
| Rabbit PPARα F | CGGCTAAAGCTGGTGTACGA |
| Rabbit PPARα R | AAAGGCACTTGTGAAAGCGG |
|  |  |
| Rabbit BDH1 F | GCTGCTTGCTGAAGGACAAG |
| Rabbit BDH1 R | CAGACATTGAGCTGGACGGT |
|  |  |
| Rabbit Acox1 F | CCGGGCAGCCAGATTAGTAG |
| Rabbit Acox1 R | GCTCGAACAAGGTCCACAGA |
|  |  |
| Rabbit CD36 F | GCTGGCTGTGTTTGGAGGTA |
| Rabbit CD36 R | TGTGCCTGTTTTCACCCAGT |
|  |  |
| Rabbit HNF4a F | ACAGATGTCCACCCCTGAGA |
| Rabbit HNF4a R | AGGGAGGCTTGACGATTGTG |
|  |  |
| Rabbit Foxa1 F | TGCTCCCCCGATAAGCTCT |
| Rabbit Foxa1 R | GTGGTTGAAGGAGTAGTGGGG |
|  |  |
| Rabbit GAPDH F | TGACGACATCAAGAAGGTGGTG |
| Rabbit GAPDH R | GAAGGTGGAGGAGTGGGTGTC |

**Table S4**. Comparison of the liver phenotypes of CF animal models (adapted from ref (1, 2)).

|  | **Main liver phenotypes** | **Key References** |
| --- | --- | --- |
| Human | Cholestasis, focal biliary cirrhosis, microgallbladder, steatosis. | (3-7) |
| Mouse | No obvious liver phonotypes. Low grade steatosis, biliary abnormalities in a small percentage of G551D mice. | (8-10) |
| Rat | No obvious liver phonotypes. | (11, 12) |
| Ferret | No obvious liver phenotypes with elevated liver enzymes (i.e., ALT, bilirubin). | (13) |
| Pig | Focal biliary cirrhosis, microgallbladder | (14, 15) |
| Sheep | Biliary fibrosis; Severe intrahepatic cholestasis | (16) |
| Rabbit | Focal biliary cirrhosis, steatosis, | Current work |

**References in the Supplementary Tables**

1. R. Fiorotto *et al.*, Animal models for cystic fibrosis liver disease (CFLD). *Biochim Biophys Acta Mol Basis Dis* **1865**, 965-969 (2019).

2. D. H. Hryciw *et al.*, Role for animal models in understanding essential fatty acid deficiency in cystic fibrosis. *Cell Mol Life Sci* **78**, 7991-7999 (2021).

3. K. Moyer, W. Balistreri, Hepatobiliary disease in patients with cystic fibrosis. *Curr Opin Gastroenterol* **25**, 272-278 (2009).

4. N. Kamal, P. Surana, C. Koh, Liver disease in patients with cystic fibrosis. *Curr Opin Gastroenterol* **34**, 146-151 (2018).

5. S. Sakiani, D. E. Kleiner, T. Heller, C. Koh, Hepatic Manifestations of Cystic Fibrosis. *Clin Liver Dis* **23**, 263-277 (2019).

6. C. Y. Ooi, P. R. Durie, Cystic fibrosis from the gastroenterologist's perspective. *Nat Rev Gastroenterol Hepatol* **13**, 175-185 (2016).

7. D. Debray *et al.*, Cystic Fibrosis-related Liver Disease: Research Challenges and Future Perspectives. *J Pediatr Gastroenterol Nutr* **65**, 443-448 (2017).

8. M. Wilke *et al.*, Mouse models of cystic fibrosis: phenotypic analysis and research applications. *J Cyst Fibros* **10 Suppl 2**, S152-171 (2011).

9. S. J. Delaney *et al.*, Cystic fibrosis mice carrying the missense mutation G551D replicate human genotype-phenotype correlations. *EMBO J* **15**, 955-963 (1996).

10. P. R. Durie, G. Kent, M. J. Phillips, C. A. Ackerley, Characteristic multiorgan pathology of cystic fibrosis in a long-living cystic fibrosis transmembrane regulator knockout murine model. *Am J Pathol* **164**, 1481-1493 (2004).

11. A. McCarron *et al.*, Phenotypic Characterization and Comparison of Cystic Fibrosis Rat Models Generated Using CRISPR/Cas9 Gene Editing. *Am J Pathol* **190**, 977-993 (2020).

12. E. Dreano *et al.*, Characterization of two rat models of cystic fibrosis-KO and F508del CFTR-Generated by Crispr-Cas9. *Animal Model Exp Med* **2**, 297-311 (2019).

13. X. Sun *et al.*, Disease phenotype of a ferret CFTR-knockout model of cystic fibrosis. *J Clin Invest* **120**, 3149-3160 (2010).

14. C. S. Rogers *et al.*, Disruption of the CFTR gene produces a model of cystic fibrosis in newborn pigs. *Science* **321**, 1837-1841 (2008).

15. A. Uc *et al.*, Pancreatic and biliary secretion are both altered in cystic fibrosis pigs. *Am J Physiol Gastrointest Liver Physiol* **303**, G961-968 (2012).

16. Z. Fan *et al.*, A sheep model of cystic fibrosis generated by CRISPR/Cas9 disruption of the CFTR gene. *JCI Insight* **3** (2018).
