# Supplementary material for: Cystic fibrosis rabbits develop spontaneous hepatobiliary lesions and CF-associated liver disease (CFLD)-like phenotypes
Source: PNAS Nexus. 2022 Dec 23;2(1):pgac306. doi: 10.1093/pnasnexus/pgac306 (PMC9832953; doi:10.1093/pnasnexus/pgac306)

**Figure S1**. Immunofluorescence staining of CFTR and CK19 in a wild-type rabbit liver serial sections. The staining reveals that the CFTR signals locate at the apical membrane of cholangiocytes where CK19, a cholangiocyte marker, is positive. DAPI staining (blue) indicates nuclei. Scale bar: 20 µm.


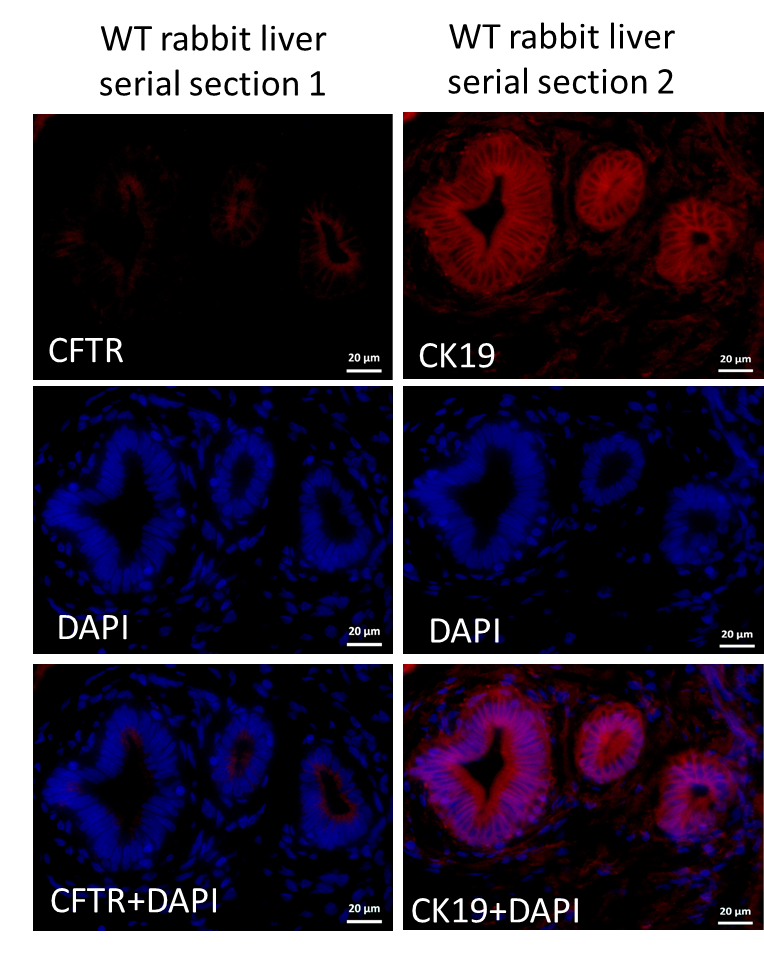


**Figure S2.** Illustration of the CF-9 mutation. The CF-9 mutation leads to the deletion of 3 amino acids (P477m, S478 and E479) in the NBD1 domain of the rabbit CFTR protein, NBD1: nucleotide-binding domain 1. R: regulatory domain. NBD2: nucleotide-binding domain 2.**
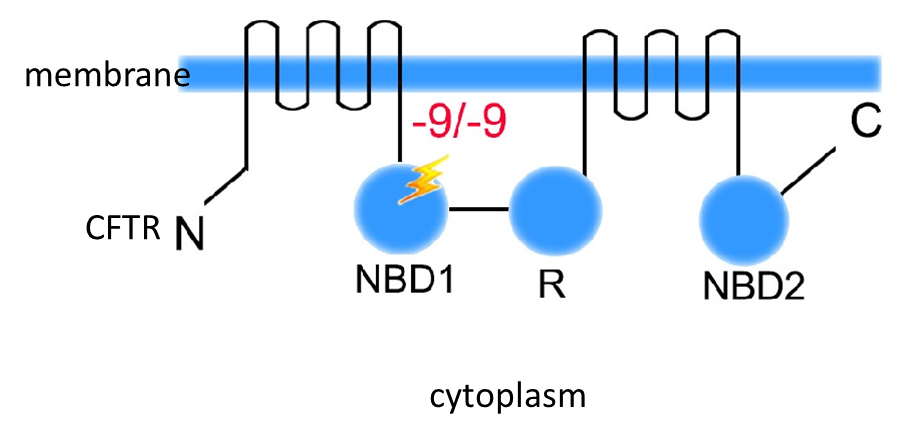
**

**Figure S3.** Illustration of CF rabbit assignments in the study. The work is conducted in two institutes: Wayne State University (WSU) and University of Michigan (UM). Experiments within each shaded box indicate that the animals used in these experiments belong to the same cohort.

**
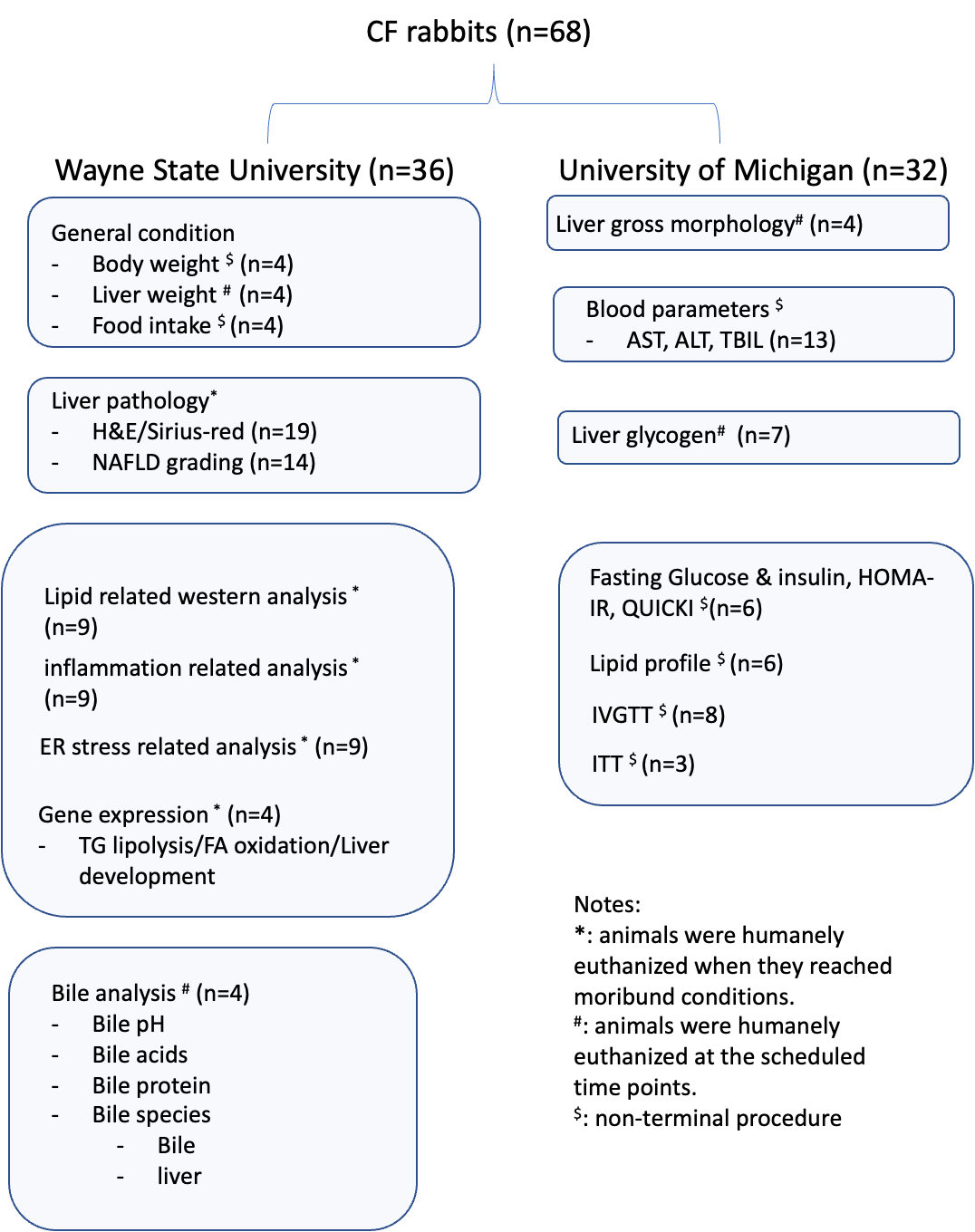
**

**Figure S4.** Inappetite scores of WT (n=4, two male, two female) and CF (n=4, two male, two female) rabbits. Each data point indicates the inappetite score of a day between the age of 43 and 49 days (i.e., Week 7) of the corresponding animal. The inappetite score is scaled from 0 to 4, with higher number indicates higher extent of inappetite. For example, “0” means a full consumption of the food, whereas “4” indicates that all food is left (i.e., none consumed).

**
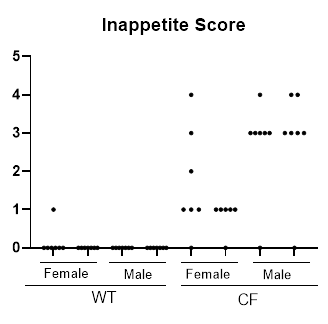
**

**Figure S5**. Morphology of bile acids from CF and WT rabbits. (A) Microscopy images of bile collected from gallbladders of CF and WT rabbits on cover-slides. Mucus and pigment stones (arrows) appeared in bile of CF rabbits. Scale bar: 200 µm. (B) Morphology of bile acids from CF, heterozygous CF (HT) and WT rabbits before shaking (left) and after shaking (right).


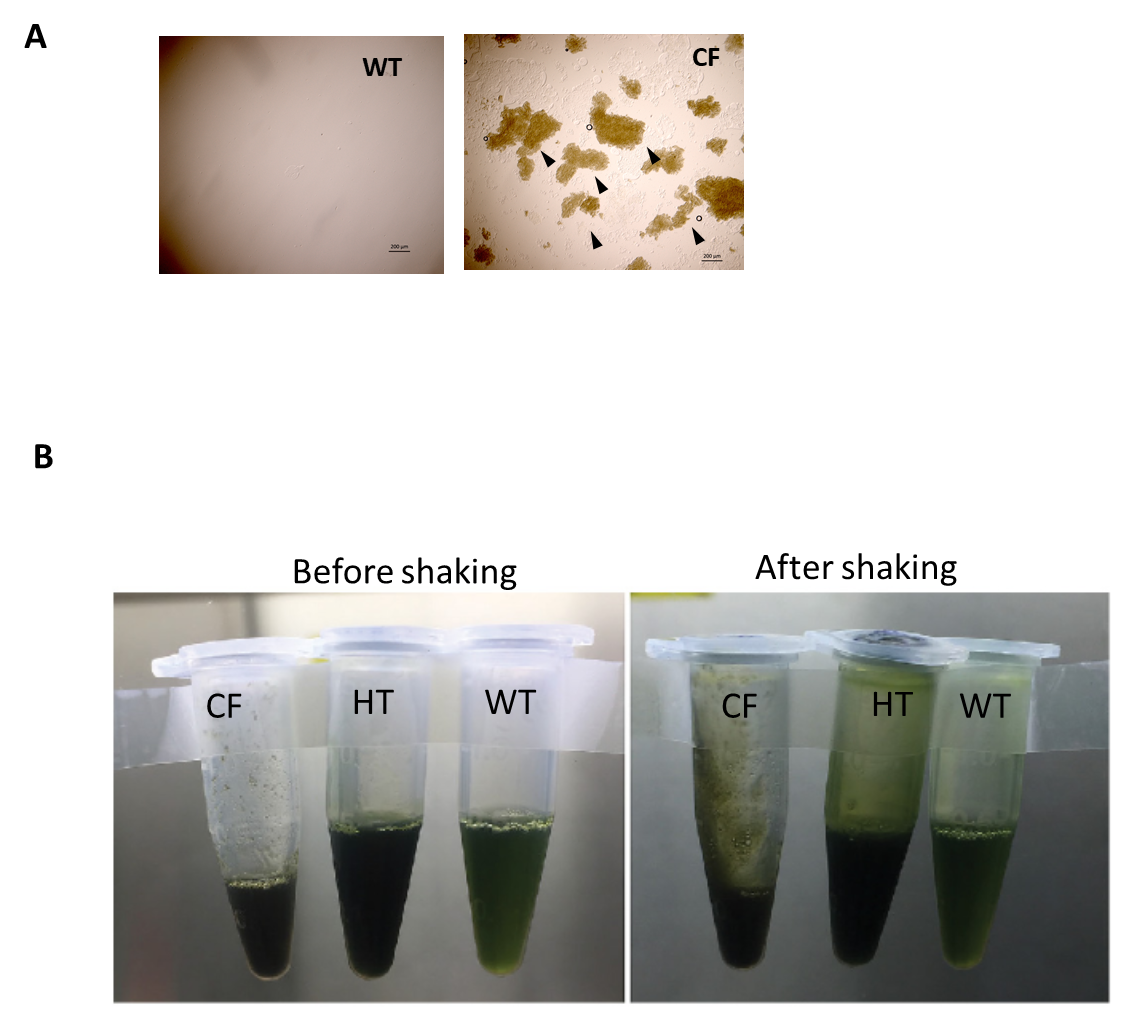


**Figure S6**. Levels of bile acid (BA) species in the liver tissue of CF rabbits. **
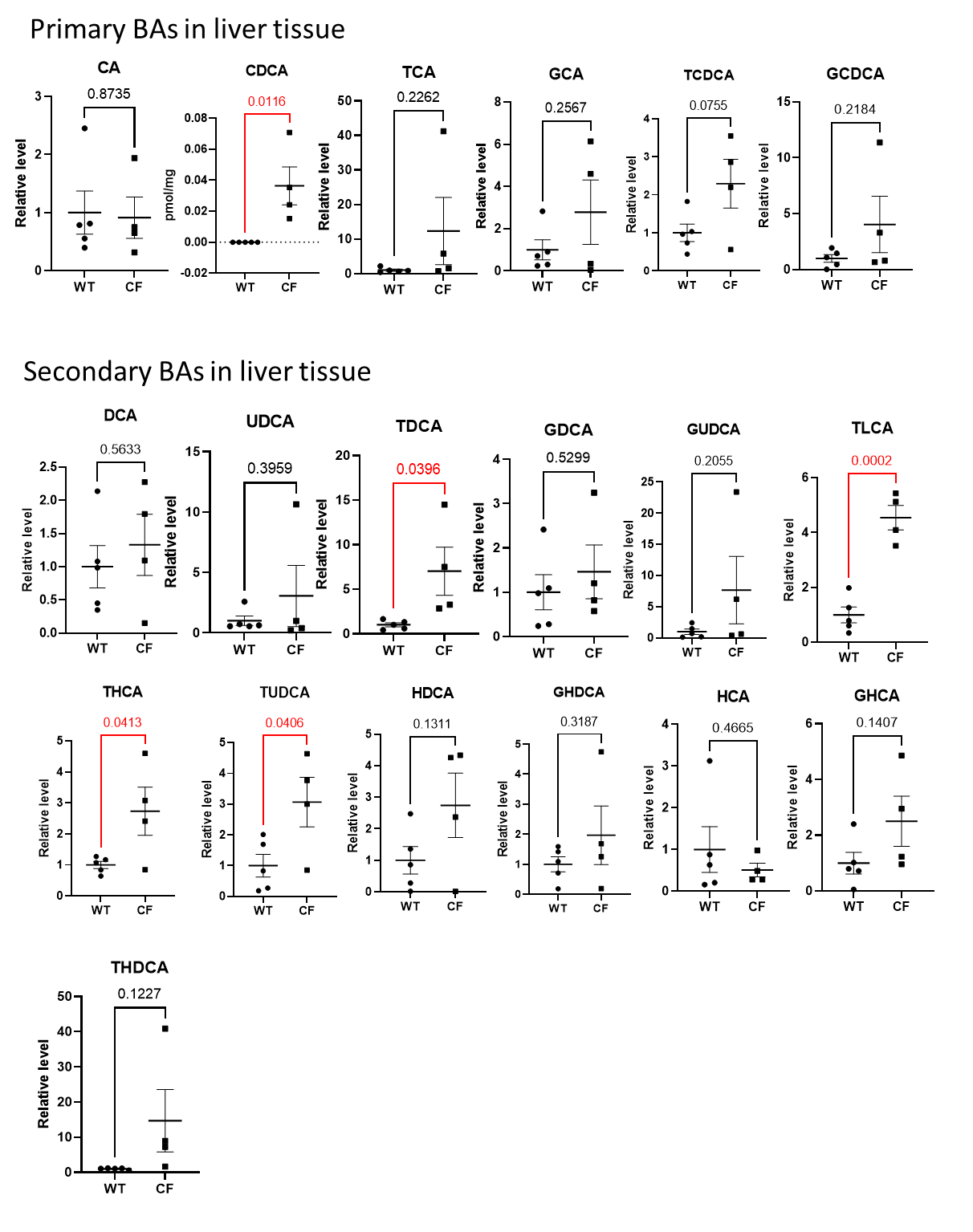
**

**Figure S7**. Levels of bile acid (BA) species in the bile of CF rabbits. **
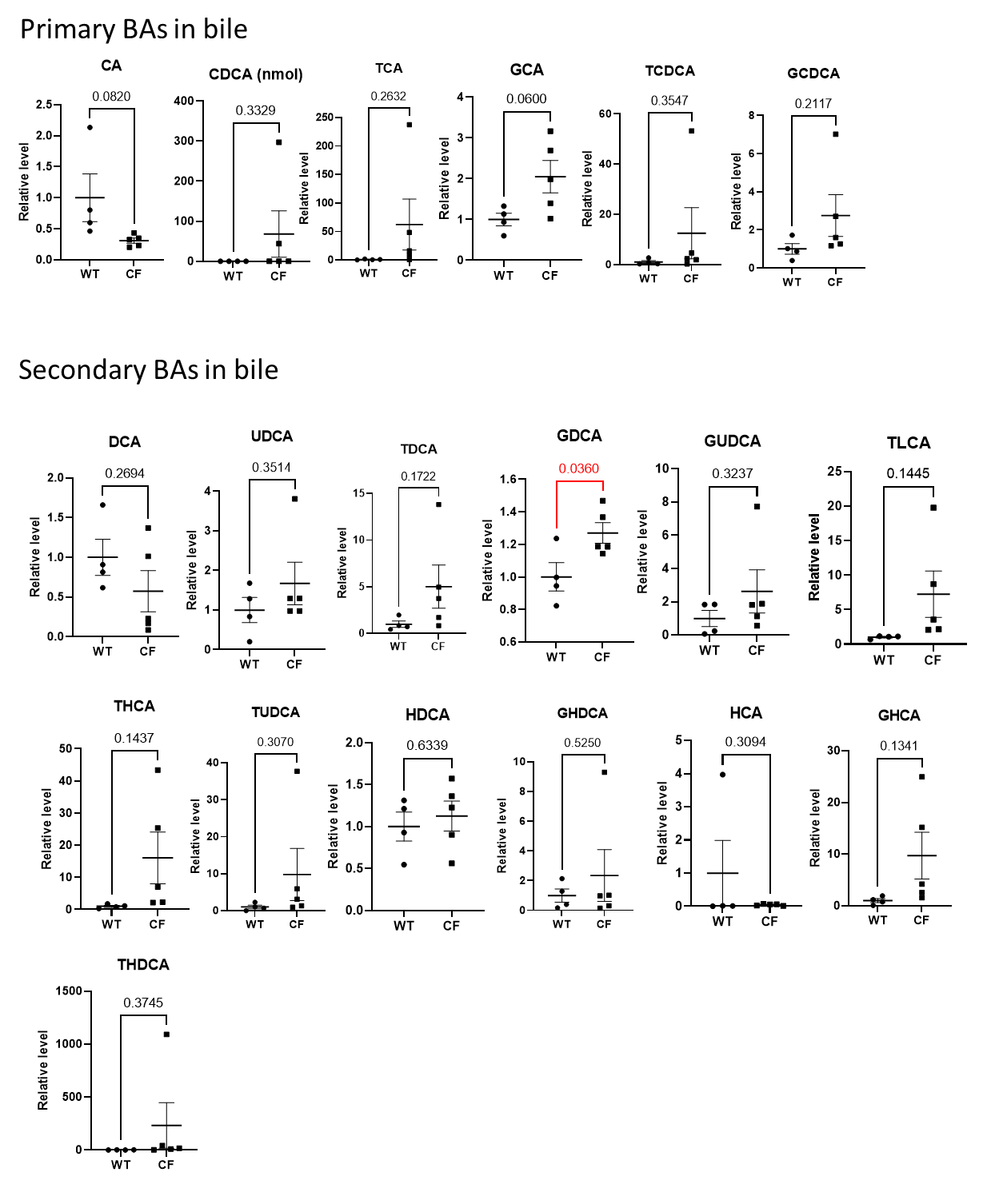
**

**Figure S8**. Total cholesterol (TC), triglyceride (TG) and free fatty acids (FFA) in the liver tissues of WT (n=6) and CF (n=6) rabbits of 7 weeks old.

P=

P=

P=

**Figure S9.** Expression of selected genes involved in (A) TG lipolysis, (B) FA oxidation, and (C) liver development in the liver of the WT (n=4, 50-70 days of age) and CF (n=4, 50-70 days of age) rabbits, determined by qPCR analyses. The average of all the control expression levels was set as 1, which was used to calculate the fold changes of all the individual expression levels.

**
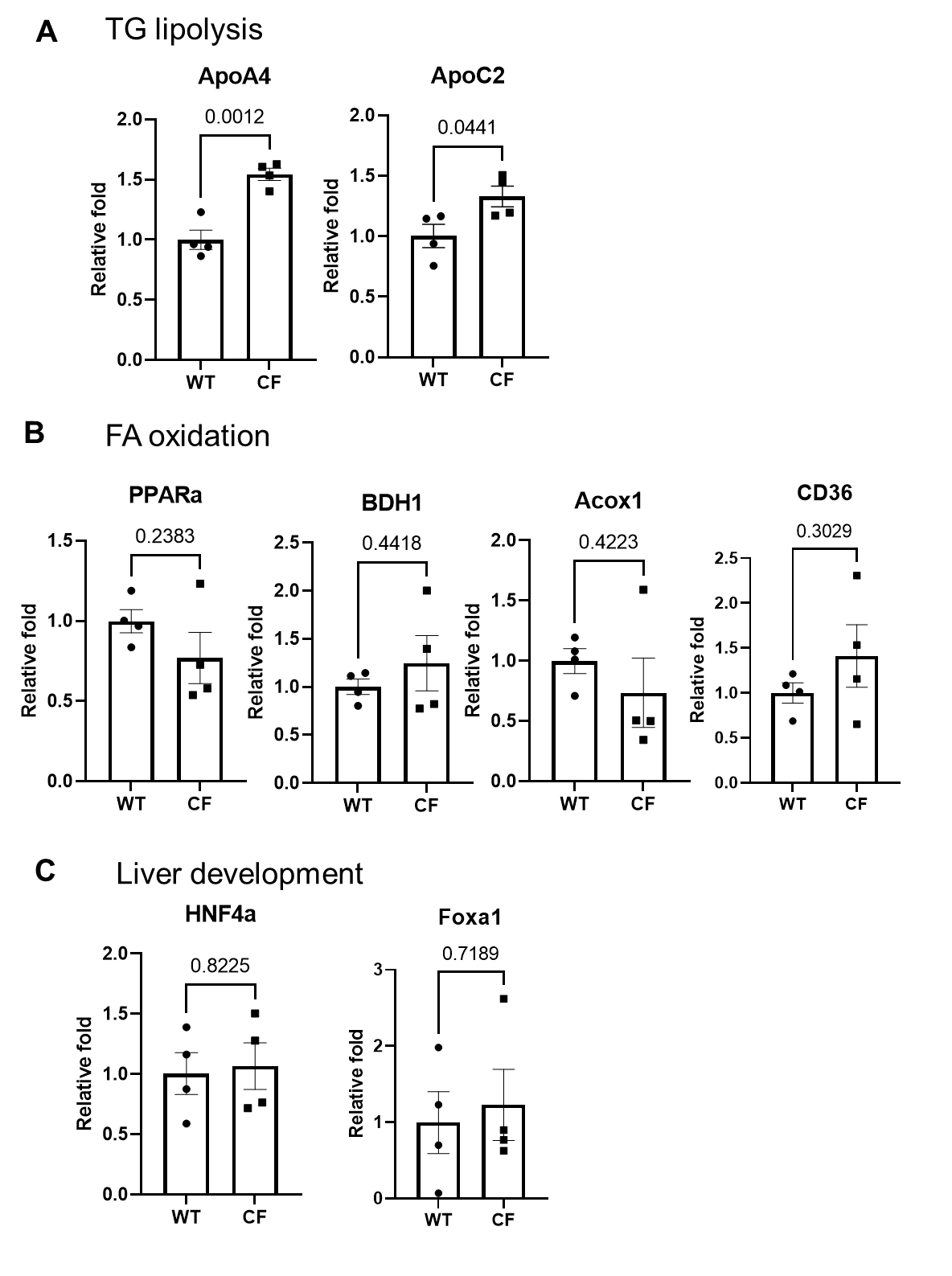
**

**Figure S10.** Glucose and insulin levels in CF rabbits. (A) Random glucose levels of CF and WT rabbits at <90 d old (left) and >=90 d old (right). (B) ITT curves of WT and CF rabbits. (C) HOMA-IR and QUICKI values of WT and CF rabbits. (D) PAS staining of hepatic glycogens in CF and WT rabbits. Scale bars: 50 μm.


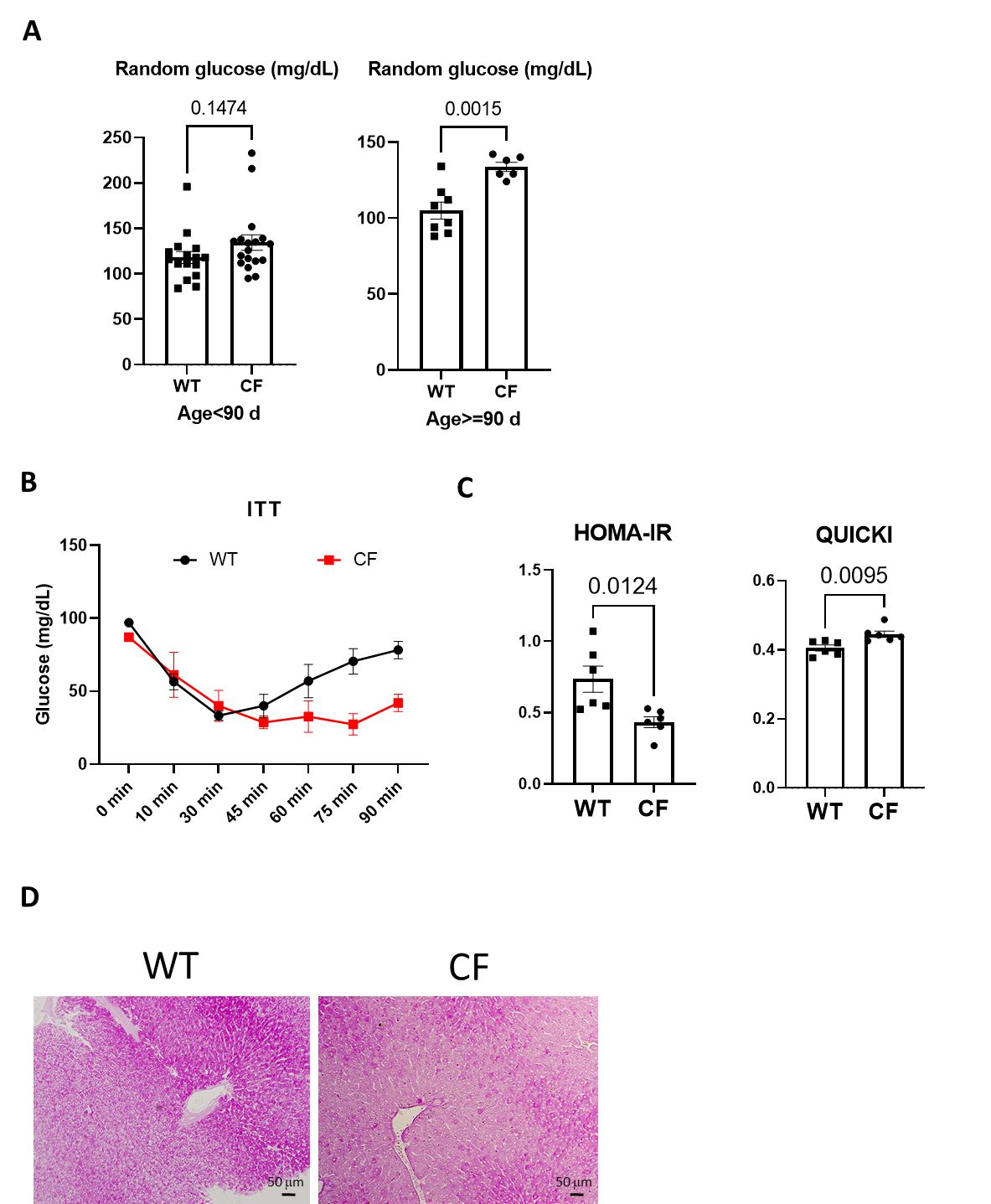

Supplement: pgac306_Supplemental_File [file pgac306_supplemental_file.zip › PNASNEXUS-PNASNEXUS-2022-00481-s02.docx]
